# Changes in perceived mental fatigue, physical fatigue and mood state during a 4‐day national junior orienteering competition preparation camp

**DOI:** 10.1002/ejsc.12071

**Published:** 2024-02-05

**Authors:** Hui Kwan Nicholas Lam, John Sproule, Anthony P. Turner, Shaun M. Phillips

**Affiliations:** ^1^ Human Performance Science Research Group The University of Edinburgh Edinburgh UK

**Keywords:** athlete wellbeing, cognitive fatigue, cross‐country, mental exertion, perception

## Abstract

Mental fatigue (MF) has been shown to acutely impair the psychological responses and endurance running performance of orienteers. This study aimed to explore MF levels experienced by orienteers during a 4‐day competition preparation camp that consisted of simulated sprint, middle‐distance, long‐distance, relay and night races. Eleven national junior orienteers participated in the study (age: 15–17 years, height: 1.69 ± 0.07 m and body mass: 59.9 ± 5.22 kg). Subjective ratings of MF, motivation, stress, physical fatigue (PF) and tiredness were measured using a 100‐mm visual analogue scale. The Brunel Mood Scale (BRUMS) was utilized to assess the mood state of the orienteers. The self‐report measures were taken within 30 min of waking, immediately after the post‐training session, and after 24 and 48 h following the final training session. The pre–post orienteering training combined analysis showed that there was a moderate increase in perceived MF (ES = 1.06 [0.66, 1.45]), PF (ES = 1.07 [0.69, 1.45]) and BRUMS fatigue (ES = 0.74 [0.4, 1.1]) after orienteering training. At 48 h post the final training session, MF remained moderately elevated (ES = 0.86 [−0.07, 1.75]), while PF also remained elevated to a small extent (ES = 0.46 [−0.46, 1.39]) compared to the pre‐training values. A moderate impairment was still observed in BRUMS vigor (ES = −1.02 [−1.65, −0.36]), but BRUMS confusion scores were moderately lower (ES = −0.85 [−1.71, 0.04]) than pre‐training values. This study found that orienteering training induced acute MF, persisting for at least 48 h after the final session.

## Highlights

- National junior orienteers experienced perceived mental fatigue during an orienteering training camp, and recovery of psychological responses was incomplete after 48 h.
- There was a moderate correlation between changes in perceived mental fatigue and physical fatigue during the orienteering training camp, suggesting that while being related they represent distinct constructs of fatigue.
- Perceived mental fatigue did not recover to its pre‐training value on the following training day, suggesting a progressive accumulation effect that persisted until the completion of the training camp.
- After the training camp, the ratings of perceived mental fatigue, stress, and physical fatigue, as well as the scores from the Brunel Mood Scale (vigor and fatigue), remained impaired for 48 h compared to their pre‐training value.

## 1 INTRODUCTION

Mental fatigue (MF) has been defined as a psychobiological state caused by performing prolonged periods of a cognitively demanding task (Van Cutsem et al., 2017). Previous systematic reviews have demonstrated that MF can detrimentally affect sport‐specific tactical performance (Cao et al., 2022; Sun et al., 2022) and endurance running performance (Brown et al., 2020; Giboin et al., 2019; Habay et al., 2021; Van Cutsem et al., 2017). The previous conceptual model of MF suggests that cognitive demand serves as a stimulus that influences the adenosine and dopamine levels in the anterior cingulate cortex (Smith et al., 2018). Consequently, this increased MF impairs executive brain function, including decision‐making ability and performance adjustment.

Orienteering requires high cognitive efforts, as orienteers must determine and execute the most efficient route to each control point in an unfamiliar environment using a map and a compass under timed conditions (Batista et al., 2020; Creagh et al., 1997). This emphasizes a simultaneous physical challenge. Specifically, Batista et al. (2021) demonstrated a slower completion time in a 3.1‐km simulated orienteering race following a 30‐min Stroop task, implying that the elevated MF can negatively affect orienteering performance. However, due to the limited ecological validity of the computerized MF protocol employed, it remains unclear whether engagement in actual orienteering activity induces perceived MF in trained orienteers. Further negative effects of MF have also been found in mood state, as studies utilizing the Brunel Mood Scale (BRUMS) have consistently shown decreased vigor and increased fatigue scores following MF elicitation (Van Cutsem et al., 2017). This suggests that an individual's mood state can indeed be affected under a state of MF, which may in turn impact on orienteering training performance. There has also been some suggestion that increased confusion may also be amongst the effects of MF in orienteering (Lam, Sproule, Turner, Murgatroyd, et al., 2023). Furthermore, it is essential to measure MF separately from other psychological responses like motivation and mood, as MF changes differ from those observed in other self‐report measures (Russell, Jenkins, Halson, Juliff, et al., 2022). Accordingly, further research is required to comprehend the presence of MF in orienteering, and subsequently validate the need for improving monitoring and management strategies to maintain or enhance performance in training and competition.

A recent Delphi study with international orienteering experts achieved consensus on the occurrence of MF during orienteering competitions (Lam, Sproule, Turner, Murgatroyd, et al., 2023). This study highlighted potential differences between MF experienced during orienteering training and competition, where the type of MF was deemed as unique within the event (Lam, Sproule, Turner, Murgatroyd, et al., 2023). For example, a 2‐year longitudinal study with elite netballers found significantly lower perceived MF ratings during competition compared to training camps and preparation camps (Russell et al., 2021). Coyne et al. (2021) found that the acute changes in perceived MF within one training session might not directly influence the perceived exertion of elite team and individual sport athletes during competition preparation, but cumulative perceived MF over a week could potentially increase the overall internal load. An observational study in elite netballers during a 16‐week preseason training phase reported significantly higher perceived MF, during the late weeks of the preseason (Russell, Jenkins, Halson, & Kelly, 2022). This research reveals that MF is not exclusive to competition, but perceived MF can also accrue throughout training. Recent research with national level orienteers found impaired subjective ratings of MF, physical fatigue (PF) and mood state following orienteering competition (Lam, Sproule, Turner, & Phillips, 2023). This supports further investigation into whether a similar phenomenon occurs in orienteering training to test the opinions of orienteering experts (Lam, Sproule, Turner, Murgatroyd, et al., 2023) and inform discussion and practice of appropriate preparation and monitoring of orienteering athletes in competition and training environments.

Therefore, this study aimed to explore the changes in perceived MF, mood state and other psychological variables amongst orienteers during an orienteering training camp. Similar to previous research conducted with elite netballers during training and competitions (Russell, Jenkins, Halson, Juliff, et al., 2022), no hypothesis was formulated in this study to enable greater flexibility in exploring potential variations and interpreting the effects of MF in orienteering.

## 2 METHODS

To reduce potential disparity between researchers' and athletes' interpretations of MF, as previous research has highlighted, the specificity of defining MF in application to the sporting context (Russell, Jenkins, Rynne, et al., 2019) and agreed MF definition was provided to the participants. The definition of MF refined by international orienteering experts: MF “an inability to maintain concentration and process information for decision‐making efficiently and effectively following a prolonged period of cognitively demanding activity” (Lam, Sproule, Turner, Murgatroyd, et al., 2023).

### 2.1 Experimental design

An observational study design with repeated measures was utilized to observe the changes in perceived MF and other psychological variables during a 4‐day orienteering competition preparation camp.

### 2.2 Participants

Eleven British national junior orienteers (5 males and 6 females, aged 15–17 years, height 1.69 ± 0.07 m, body mass 59.9 ± 5.22 kg and 8.7 ± 3.8 years competitive experience; England: *n* = 7 and Scotland: *n* = 4) participated. All participants were full‐time students and were on spring break when the research was conducted in their places. One participant did not complete the second and the third day of the survey due to technical issues with data collection. The data from this participant were included in the analysis, except for the second and the third day of the orienteering training camp. All orienteers were classified as Tier 3 highly trained individuals (McKay et al., 2022); they were also all capable of reading and writing English fluently. Following institutional ethical approval, written informed consent and parental consent were obtained prior to the experiment. According to the precision planning for a paired‐sample design using the Exploratory Software for Confidence Intervals (Cumming, 2012), a sample size of 17 was needed to achieve an average target margin of error of 0.4 using *ρ* = 0.70 to achieve the desired level of precision. The sample size obtained in this study provides a target margin of error of 0.55 for a 95% confidence interval.

### 2.3 Orienteering training camp

The 4‐day orienteering training camp was designed by British Orienteering Talent Squad coaches. Participants arrived at the training venue 1 day before the training camp commenced, and all resided in the same accommodation throughout the camp. The primary objective of the camp was designed to replicate the orienteering competition conditions including relay, sprint and middle‐ and long‐distance race, to prepare orienteers for upcoming competition. All participants followed an identical training program, with the main training sessions lasting approximately 6 h, plus an additional hour allocated for pre‐race preparation and another hour for post‐race analysis. The only exception was on day 4 where all participants were trained for approximately 4 h only. The race events included the following: middle‐distance and sprint distance races on day 1, a long‐distance race on day 2, the second and third legs of the relay, and night orienteering on day 3, followed by a mass start relay on day 4.

### 2.4 Procedure

An online self‐report questionnaire was completed using the Qualtrics XM Platform (USA). The self‐report measures were completed daily, within 30 min of waking on each of the 4 training days (PRE1‐4) and within 30 min of completing each training session across the 4 training days (POST1‐4), similar to prior research (Lam, Sproule, Turner, & Phillips, 2023; Russell, Jenkins, Halson, Juliff, et al., 2022). To explore post‐camp recovery responses, participants completed self‐report measures 24 (24POST) and 48 h (48POST) after the training camp. The self‐report measures were completed privately on participants' own electronic devices such as laptops, smartphones or tablets to reduce the response bias (Russell, Jenkins, Halson, & Kelly, 2022).

### 2.5 Online self‐report measures

The daily self‐report measurements contained 18 items, including a closed‐ended question: “Did you compete in an orienteering training session just now?”. Five items were measured via 100‐mm visual analogue scales (VAS) (ranging from 0 to 100) including MF, motivation, PF, stress and tiredness. A further 12 items from the vigor, fatigue and confusion subscales of the Brunel Mood Scale (BRUMS) were measured using a 0–4 Likert scale (Brandt et al., 2016). Additionally, six items regarding the participant's characteristics, such as age, gender, height, body mass, geographical location and years of experience, competing in orienteering were collected at completion of the PRE1 questionnaire. All participants completed the identical self‐report measures 1 week prior to the experiment as a familiarization trial.

### 2.6 Perceived mental fatigue, physical fatigue, motivation, tiredness and stress

The 100‐mm VAS have demonstrated a high reliability in quantifying perceived MF and fatigue‐related symptoms (Lee et al., 1991; Smith et al., 2019). Similar online observational research conducted with academy soccer players, also measured perceived MF using a 100‐mm VAS (Thompson et al., 2020), which has been identified as the most appropriate approach for assessing subjective MF for field‐based research (Smith et al., 2019). Given the subjective nature of MF and other outcome variables, this study also employed VAS to measure other outcome variables. The questions and descriptors for the five variables measured using the 0–100 sliding scale were identical to Lam, Sproule, Turner, & Phillips, 2023). In consideration of the complexity and potentially varying interpretations of MF as highlighted in previous research (Russell, Jenkins, Rynne, et al., 2019), Russell, Jenkins, Halson, & Kelly, 2019nd to minimize any potential confusion between MF and other outcome variables, participants were provided with a modified definition of MF by international orienteering experts (Lam, Sproule, Turner, Murgatroyd, et al., 2023) when completing the 100‐mm VAS.

### 2.7 Mood state

The vigor and fatigue subscales of BRUMS have been shown to be negatively affected by MF (Van Cutsem et al., 2017), and international orienteering experts have highlighted the importance of measuring confusion when evaluating the mood state of mentally fatigued orienteers (Lam, Sproule, Turner, Murgatroyd, et al., 2023). Therefore, participants rated 12 items on a 0–4 Likert scale regarding vigor, fatigue and confusion subscales of the BRUMS by answering a standardized question: “How do you feel right now” (Brandt et al., 2016). Each item was scored from 0 to 4 (0 = not at all, 1 = a little, 2 = moderately, 3 = quite a bit and 4 = extremely). The four relevant items from each subscale were summated to obtain a score between 0 and 16.

### 2.8 Statistical analysis

Data were presented as mean ± SD unless specified. It has been criticized that null‐hypothesis testing on small sample sizes can potentially lead to an overestimation of results (Batterham et al., 2006). Additionally, it is important to note that interpreting *p*‐value alone does not provide information about the magnitude or direction of changes in measurements. Therefore, this study analyzed the changes using effect size (ES) with 95%CI. The magnitude of change was measured for the subjective ratings of MF, motivation, PF, stress, tiredness and BRUMS. The following equation was used to calculate the ES in this study:

$$d=\frac{{\bar{x}}_{1}-{\bar{x}}_{2}}{\sqrt{\frac{s_{1}^{2}+s_{2}^{2}}{2}}}$$

The ES was interpreted trivial (0.00–0.19), small (0.20–0.59), moderate (0.60–1.19), large (1.20–1.99), very large (2.00–3.99) and extremely large (≥4) (Batterham et al., 2006; Hopkins et al., 2009). If the 95%CI of the ES does not overlap with the value of 0, it can be deemed as statistically significant. To explore the effect of an orienteering training day, all outcome variables were compared pairwise before (PRE values on days 1–4) and after training each day (POST values on days 1–4). To explore recovery, the responses changes immediately after POST4, 24 (24POST) and 48 h after (48POST) the last training session of the training camp were compared. The relationship between the pre‐ and post‐ orienteering training combined changes in MF and PF were assessed via Pearson's correlation coefficient. The correlation coefficients were interpreted as: trivial (*r* < 0.1), small (*r* ≤ 0.29), moderate (*r* ≤ 0.49), large (*r* ≤ 0.69), very large (*r* ≤ 0.89), nearly perfect (*r* ≤ 0.99) and perfect (*r* = 1.0).

## 3 RESULTS

### 3.1 Pre‐ and post‐ orienteering training

**FIGURE 1:**
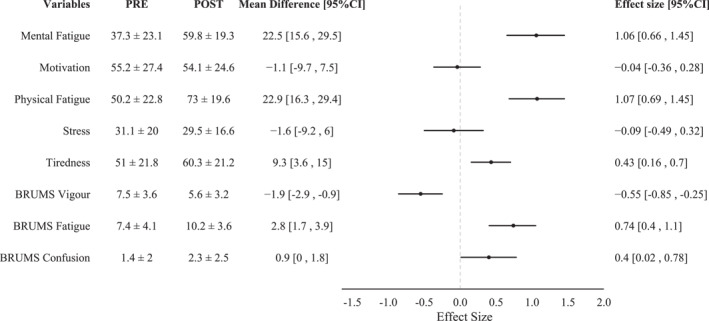
The magnitude of changes in all outcome variables before and after an orienteering training. Data has been combined from all participants (42 responses).

The analysis in this section included the combination of all pre–post measures of the outcome variables from day 1 to day 4 of the training camp. The mean pre–post orienteering training change in all outcome variables for all participants is presented in Figure 1. There was a moderate increase in subjective ratings of MF, PF and BRUMS fatigue score and a small increase in tiredness and BRUMS confusion score following orienteering training. Conversely, there was a small decrease in BRUMS vigor scores and a trivial decline in motivation and stress ratings after orienteering training. A moderate correlation was observed between the pre‐ and post‐orienteering training changes in MF and PF (*r* = 0.47 [0.18, 0.67]).

### 3.2 Pre‐to post‐training changes across consecutive days

The data for changes in survey responses across the 4 consecutive days of training are displayed in Figure 2, and the difference in the extent of changes between each training day of 10 participants is in Table 1. One of the participants failed to complete the survey on day 2 and day 3 of the orienteering training camp due to technical difficulties. Figure 2 shows that the ratings of perceived MF, PF and BRUMS fatigue before orienteering training progressively increased across the four training days, with a moderate increase observed in the pre‐training value of MF (PRE1 vs. PRE2: ES = 0.74 [−0.02, 1.46]) and PF (PRE3 vs. PRE4: ES = 0.64 [−0.08, 1.32]). However, the other outcome variables did not demonstrate a consistent increasing or decreasing trend during the training camp. As for the post orienteering training ratings, only the rating of MF presented a constant increase with a trivial to small ES (POST1 vs. POST2: ES = 0.49 [−0.18, 1.13]; POST2 vs. POST3: ES = 0.11 [−0.37, 0.58]; and POST3 vs. POST4: ES = 0.44 [0.05, 0.82]). The remaining outcome variables did not exhibit a consistent pattern of increase or decrease.

FIGURE 2Changes in MF (A), motivation (B), PF (C), stress (D), tiredness (E), BRUMS vigor subscale (F), BRUMS fatigue subscale (G), and BRUMS confusion subscale (H) from day 1–48 h after the orienteering training camp. The gray lines represent individual ratings of the 10 participants. For time points PRE2, PRE3, POST2 and POST3, the mean value of responses from 10 participants was used, while for the remaining time points, all participants (*n* = 11) were included.

**Figure:**
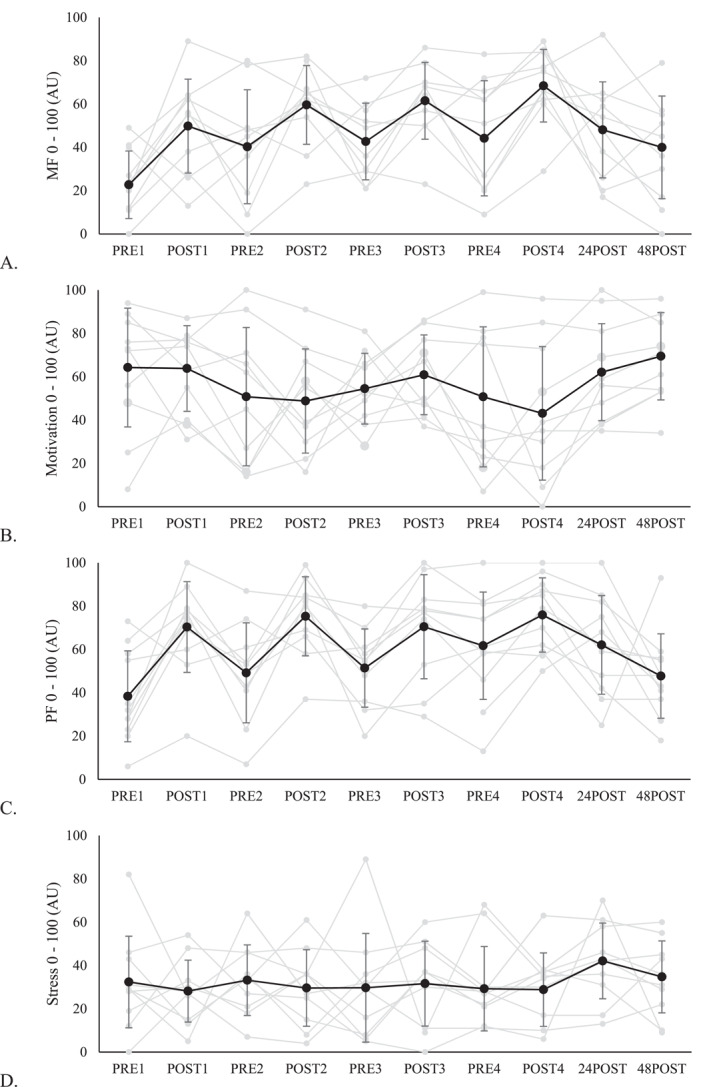

**Figure:**
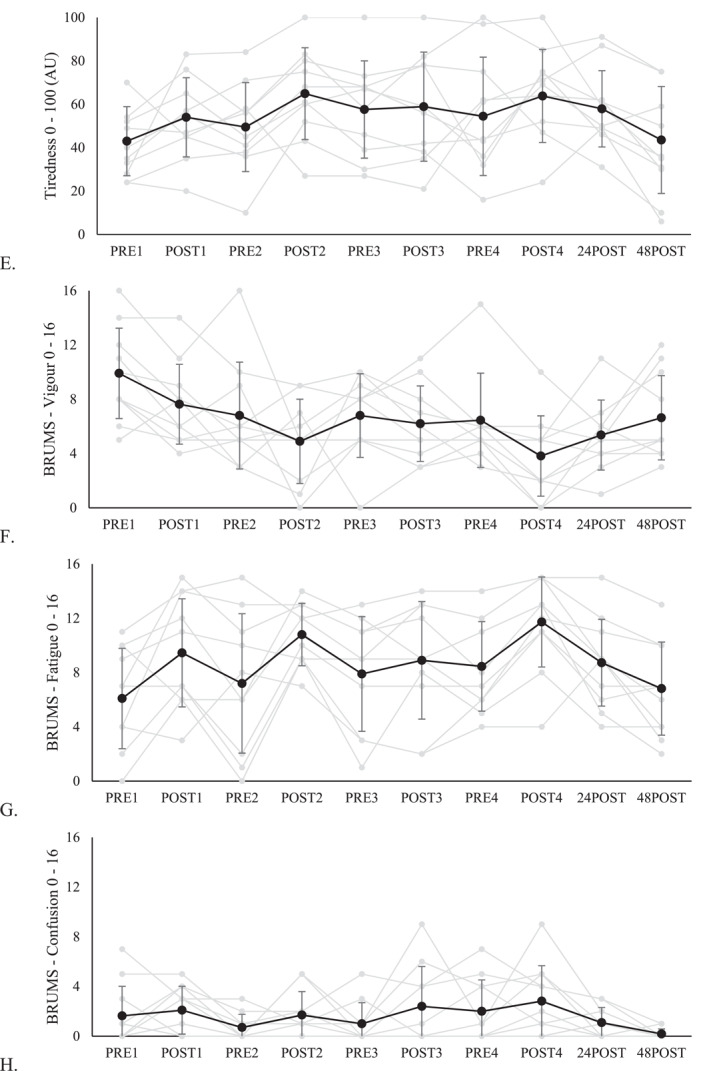

**TABLE 1:** The comparison of the magnitude of changes in all outcome variables before and immediately after training from 10 participants.

Variables	PRE versus POST				
	Mean difference [95%CI]	Effect size [95%CI]	Changes between days (effect size [95%CI])		
			vs day 2	vs day 3	vs day 4
Mental fatigue 0–100 AU					
Day 1	25 [4, 46]	1.29 [0.16, 2.37]^b^	−0.21 [−1.06, 0.66]	−0.26 [−0.84, 0.34]	−0.13 [−0.89, 0.64]
Day 2	19.3 [0.8, 37.8]	0.85 [0.03, 1.64]^a^		−0.02 [−0.64, 0.6]	0.13 [−0.56, 0.81]
Day 3	18.8 [6.1, 31.5]	1.06 [0.26, 1.83]^a^			0.19 [−0.42, 0.79]
Day 4	22 [10.8, 33.2]	1 [0.35, 1.62]^a^			
Motivation 0–100 AU					
Day 1	−0.6 [−18.5, 17.3]	−0.03 [−0.66, 0.61]	−0.05 [−0.64, 0.55]	0.28 [−0.44, 0.98]	−0.12 [−1.07, 0.84]
Day 2	−2 [−25.1, 21.1]	−0.07 [‐0.78, 0.64]		0.29 [−0.45, 1.01]	−0.06 [−0.89, 0.78]
Day 3	6.4 [−11.6, 24.4]	0.37 [−0.56, 1.27]			−0.37 [−0.98, 0.25]
Day 4	−3.8 [−24.8, 17.2]	−0.12 [−0.68, 0.45]			
Physical fatigue 0–100 AU					
Day 1	30.9 [11.4, 50.4]	1.4 [0.38, 2.38]^b^	−0.19 [−0.91, 0.55]	−0.53 [−1.4, 0.38]	−0.85 [−1.78, 0.12]^a^
Day 2	26.1 [8.7, 43.5]	1.25 [0.31, 2.15]^b^		−0.34 [−1.17, 0.51]	−0.68 [−1.64, 0.31]^a^
Day 3	19.1 [7.4, 30.8]	0.9 [0.26, 1.51]^a^			−0.43 [−1.47, 0.64]
Day 4	12.8 [3.4, 22.2]	0.62 [0.12, 1.09]^a^			
Stress 0–100 AU					
Day 1	−4 [−23.9, 15.9]	−0.21 [−1.12, 0.71]	0.02 [−0.62, 0.65]	0.19 [−0.47, 0.84]	0.15 [−0.72, 1.01]
Day 2	−3.6 [−18.4, 11.2]	−0.21 [−0.97, 0.55]		0.2 [−0.58, 0.97]	0.16 [−0.96, 1.28]
Day 3	1.9 [−21.7, 25.5]	0.08 [−0.83, 1]			−0.08 [−0.77, 0.61]
Day 4	−0.3 [−14.7, 14.1]	−0.02 [−0.71, 0.67]			
Tiredness 0–100 AU					
Day 1	11.9 [−3, 26.8]	0.7 [−0.14, 1.5]^a^	0.17 [‐0.56, 0.9]	−0.64 [−1.53, 0.27]^a^	−0.11 [−0.98, 0.76]
Day 2	15.4 [1.4, 29.4]	0.74 [0.05, 1.4]^a^		−0.89 [−1.83, 0.09]^a^	−0.28 [−1.23, 0.68]
Day 3	1.3 [−6.3, 8.9]	0.06 [−0.23, 0.33]			0.48 [−0.57, 1.5]
Day 4	9.5 [−6.2, 25.2]	0.39 [‐0.2, 0.95]			
BRUMS vigor 0–16					
Day 1	−2.2 [−4, −0.4]	−0.67 [−1.22, −0.09]^a^	0.08 [−0.64, 0.79]	0.64 [−0.47, 1.71]^a^	−0.04 [−0.82, 0.74]
Day 2	−1.9 [−5.3, 1.5]	−0.54 [−1.4, 0.35]		0.34 [−0.6, 1.26]	−0.11 [−0.79, 0.58]
Day 3	−0.6 [−2.4, 1.2]	−0.2 [−0.74, 0.34]			−0.73 [−1.87, 0.44]^a^
Day 4	−2.3 [−3.8, −0.8]	−0.72 [−1.23, −0.18]^a^			
BRUMS fatigue 0–16					
Day 1	3.1 [0.1, 6.1]	0.78 [0.01, 1.51]^a^	0.11 [−0.86, 1.08]	−0.58 [−1.43, 0.3]	0.03 [−0.99, 1.05]
Day 2	3.6 [0.3, 6.9]	0.9 [0.05, 1.72]^a^		−0.68 [−1.52, 0.2]^a^	−0.11 [−1.1, 0.88]
Day 3	1 [−1, 3]	0.23 [−0.2, 0.65]			0.92 [−0.06, 1.85]^a^
Day 4	3.2 [1.9, 4.5]	0.92 [0.38, 1.44]^a^			
BRUMS confusion 0–16					
Day 1	0.5 [−1.5, 2.5]	0.23 [−0.57, 1.01]	0.2 [−0.76, 1.15]	0.3 [−0.79, 1.37]	0.13 [−0.93, 1.18]
Day 2	1 [−0.6 2.6]	0.65 [−0.33, 1.6]^a^		0.14 [−0.27, 0.55]	−0.04 [−0.86, 0.79]
Day 3	1.4 [−0.9, 3.7]	0.55 [−0.3, 1.36]			−0.15 [−0.88, 0.58]
Day 4	0.9 [−1.4, 3.2]	0.33 [−0.44, 1.09]			

^a^ A moderate difference (*d* ≥ 0.6).

^b^ A large difference (*d* ≥ 1.2).

### 3.3 Changes after the termination of the orienteering training camp

Figure 2 also presented the mean change and individual ratings of all participants (*n* = 11) immediately (POST4), 24 and 48 h after the final day of the orienteering training camp, with the magnitude of changes summarized in Table 2. From POST4 to 48POST, there was a consistent improvement in the majority of the variables. There was a progressive increase in the ratings of motivation and BRUMS vigor, and a constant decrease in MF, PF, tiredness, BRUMS fatigue and confusion scores. The rating of PF showed a moderate decrease in both POST4 to 24POST and 24POST to 48POST. However, stress rating was the only variable that increased moderately from POST4 to 24POST but declined to a small extent from 24POST to 48POST. Consequently, ratings of MF were higher, and BRUMS vigor had lower to a moderate extent in 48POST compared to PRE1.

**TABLE 2:** The extent of the changes in all outcome variables immediately and 24 and 48 h after the last training session of the orienteering training camp.

Variables	POST4 versus 24POST		24POST versus 48POST		PRE1 versus 48POST	
	Mean difference [95%CI]	Effect size [95%CI]	Mean difference [95%CI]	Effect size [95%CI]	Mean difference [95%CI]	Effect size [95%CI]
MF	−20.4 [‐40.5, −0.2]	−1.04 [−2.02, −0.01]^a^	−8.1 [−22.3, 6.1]	−0.35 [−0.91, 0.22]	17.3 [−1.7, 36.3]	0.86 [−0.07, 1.75]^a^
Motivation	19 [7.2, 30.8]	0.71 [0.2, 1.19]^a^	7.4 [−0.6, 15.4]	0.35 [−0.02, 0.7]	5.2 [−13.6, 23.9]	0.22 [−0.48, 0.9]
PF	−13.8 [−27.6, 0]	−0.69 [−1.34, 0]^a^	−14.4 [−36.7, 8]	−0.68 [−1.64, 0.31]^a^	9.4 [−11.8, 30.6]	0.46 [−0.49, 1.39]
Stress	13.3 [−0.7, 27.2]	0.77 [−0.03, 1.54]^a^	−7.4 [−21.9, 7.2]	−0.43 [−1.2, 0.35]	2.4 [−11.7, 16.5]	0.12 [−0.53, 0.78]
Tiredness	−5.9 [−19.3, 7.5]	−0.3 [−0.91, 0.32]	−14.4 [−26, −2.7]	−0.67 [−1.22, −0.1]^a^	0.5 [−13.5, 14.6]	0.03 [−0.57, 0.62]
Vigor	1.5 [−0.5, 3.6]	0.56 [−0.15, 1.24]	1.3 [−0.7, 3.2]	0.45 [−0.2, 1.07]	−3.3 [−5, −1.5]	−1.02 [−1.65, −0.36]^a^
Fatigue	−3 [−5.3, −0.7]	−0.92 [−1.64, −0.17]^a^	−1.9 [−3.4, −0.4]	−0.58 [−1.03, −0.1]	0.7 [−2.2, 3.6]	0.2 [−0.52, 0.92]
Confusion	−1.7 [−3.5, 0]	−0.79 [−1.54, 0]^a^	−0.9 [−1.7, −0.1]	−1 [−1.89, −0.08]^a^	−1.5 [−3, 0.1]	−0.85 [−1.71, 0.04]^a^

*Note*: Data from all participants (*n* = 11).

^a^ Moderate extent of change in the comparison (ES ≥ 0.6).

## 4 DISCUSSION

This study examined the impact of an orienteering training camp on perceived MF and other psychological variables in trained orienteers. A moderate acute increase in perceived MF was found after orienteering training, and the elevated ratings did not return to Day 1 pre‐training (PRE1) values on the subsequent training day, with the highest perceived MF being reported after training on the fourth day of the camp, suggesting a progressive accumulation effect. The elevated MF after participating in a 4‐day orienteering camp did not return to PRE1 values even 48 h after the training camp. A moderate correlation was also noted between changes in PF and MF.

### 4.1 Acute changes in perceived mental fatigue after orienteering training

The combined pre–post orienteering training session changes analysis revealed a moderate increase in perceived MF, which supports the orienteering expert's consensus in Lam, Sproule, Turner, Murgatroyd, et al., 2023) that orienteers experience MF during an orienteering training session. The data also support the findings with elite netballers where participating in sport could acutely induce MF (Russell, Jenkins, Halson, & Kelly, 2019). However, how much a moderate increase in perceived MF induced by orienteering itself affects subsequent orienteering performance remains unclear. While Batista et al. (2021) reported a trivial decrease (ES: 0.20) in 3.1‐km completion time for mentally fatigued orienteers, it is important to note that the MF was induced through a 30‐min cognitively demanding task, which has been criticized for its low ecological validity (Van Cutsem et al., 2017). The MF protocol with higher ecological validity, conducted by Coutinho et al. (2017), found a moderate increase in perceived MF (ES = 0.6) and a reduced ability to utilize environmental information following 20 min of physical activity, which affected the tactical performance of soccer players. In this study, all participants adhered to an identical training program and engaged in various types of races each day during the orienteering competition preparation camp. It is essential to clarify that this study did not compare or analyze the influence of MF on orienteering performance directly. However, given the impairment of MF ratings and subsequent soccer performance observed in previous research using a combined physical and cognitive load MF protocol (Coutinho et al., 2017), the ES obtained in this study suggests that future research could consider using orienteering training as an MF elicitation method to investigate its impact on orienteering decision‐making performance. This study acknowledges a wide confidence interval of the ES in the combined analysis, ranging from 0.66 to 1.45. However, the lower limit of the ES is still above 0.6, suggesting that there is likely to be a moderate impact on orienteering performance, as the ability to utilize environmental information is crucial for navigational ability.

This study also highlighted the consecutive changes in perceived MF throughout the 4‐day orienteering training camp, revealing a delayed recovery where the perceived MF did not return to the similar level as the pre‐training value from day 1–4. In addition to a progressively accumulating effect, we were unfortunately unable to determine any specific influence of race distance on MF during the training camp because participants undertook races within a single day. It remains debatable whether such a phenomenon appears in multiple days of actual orienteering competition as a recent review has reported that the extent of MF is related to the amount of effort invested (Giboin et al., 2019) and orienteering experts have concluded the type of MF was perceived to be different between training and competition (Lam, Sproule, Turner, Murgatroyd, et al., 2023). The competition preparation camp in this study had different conditions compared to an actual competition, including variations in atmosphere, duration and psychological responses. The study design meant that all athletes stayed in the same accommodation and followed very similar daily patterns. However, it is still likely that there were inter‐individual differences in other factors that may influence recovery. For example, previous research has reported the relevance of sleep for MF (Loch et al., 2020), yet sleep was not monitored in the current training camp. Importantly, the participants arriving at the venue a day before the start of the training camp may serve as a confounding variable in assessing the overall recovery. Previous research has reported that the sleep quality of elite athletes is negatively affected by the “first‐night effect” (Hof zum Berge et al., 2021). The changes in sleeping environment and activity patterns may contribute to reduced mental recovery (Loch et al., 2020), and the intense training schedule could further increase the challenges in determining whether the changes are related to training and/or poor recovery. Therefore, it is advisable to measure sleep quality when examining the overall recovery of athletes in future research. As the ratings of MF are subjective, the findings only reflect changes in psychological responses specifically during the competition preparation camp and may not be generalized to other occasions, such as different training camps or actual competitions. The amount of time needed to recover from MF acutely elicited by participating in sport is unclear as previous observational studies with soccer players (Thompson et al., 2020; Abbott et al., 2020) and netball players (Russell, Jenkins, Halson, & Kelly, 2019) have demonstrated that MF can impair recovery.

### 4.2 Acute changes in other psychological variables and mood state after orienteering training

Even though a moderate correlation between the pre‐ and post‐ orienteering changes in perceived MF and PF was reported, the pattern of changes between MF and PF before and after orienteering training was different, particularly on post‐training value on day two to three of the training camp. Furthermore, a variance of 22.1% (R‐squared) clearly illustrates that although MF and PF were somewhat related, the athletes consider them to be distinct phenomena. The ratings of MF constantly increased after each training session, indicating a progressive increase in MF. This supports the findings in previous research with elite netball players (Russell et al., 2019b, 2022a), indicating MF is a largely separate construct from PF and should be managed intentionally. It is important to emphasize that the goal of the training camp is to purposefully increase the amount of stress and training load in order to support athletes' physiological adaptability and development. While orienteering training acutely increased perceived MF and PF, which may have affected the athletes' overall psychological responses, future longitudinal studies will be insightful in determining how these acute changes that occur during the training camp affect the athletes' overall training adaptation accumulated over time. In contrast, a trivial to small decline in stress ratings following orienteering training reflects that perceived stress is unlikely to be related to MF. This observation supports the findings of a 2‐year longitudinal study with elite netballers where stress was reported to be different to MF (Russell, Jenkins, Halson, Juliff, et al., 2022). The higher stress ratings before training amongst the participants in this study, who were performing a simulated orienteering race, may be attributed to pre‐competition stress as indicated by the international orienteering experts (Lam, Sproule, Turner, Murgatroyd, et al., 2023). When comparing the extent of changes between different training days, it is important to acknowledge the wide confidence interval, reflecting the greater variability in the data. This variability is justifiable due to the nature of subjective measures, which can vary between individuals. The same applies to other outcome variables, where the confidence intervals of the findings overlapped with the value of 0. This indicates that the observed differences are not large enough to report meaningful changes, and there is no statistically significant difference. Moreover, the trivial changes in stress ratings can be explained by the nature of orienteering, as orienteers are used to performing under a high physical and cognitive stress environment (Batista et al., 2020; Eccles et al., 2015). In support of this, Martin et al. (2019) have found that the individuals who frequently engage in a high self‐regulated environment perceive less stress than others. Similar to previous research, the perceived stress measured in this study was not clearly defined where there might be a variation in the interpretation and definition of stress between participants (Lam, Sproule, Turner, & Phillips, 2023; Russell, Jenkins, Halson, Juliff, et al., 2022). For a more accurate measurement of stress, future research should specify the type of stress, for example, cognitive stress instead of a general stress in order to further explore any potential interplay between stress and MF.

Although a trivial to moderate increase in the ratings of tiredness was reported after orienteering training, the extent of changes was different between MF and tiredness which highlighted that MF should not be defined as an increase in perceived tiredness. This is in agreement with previous data in elite netball players (Russell, Jenkins, Halson, Juliff, et al., 2022). In agreement with the revised definition of MF mentioned in Lam, Sproule, Turner, Murgatroyd, et al., 2023), the difference in the changes between MF and tiredness ratings in the current study challenge the accuracy of the existing definition of MF that is widely used in the literature (Habay et al., 2021; Van Cutsem et al., 2017). Accordingly, it may be reasonable for future investigation with orienteers to utilize the revised definition of MF to enhance the specificity and accuracy of the discussion.

As reported in the systematic review by Van Cutsem et al. (Van Cutsem et al., 2017), the vigor and fatigue ratings of BRUMS are negatively affected when individuals experience MF. In the current study, we consistently observed that ratings of BRUMS vigor decreased, and fatigue increased, when the participants reported a higher perceived MF after each training session. However, we utilized an observational study design, meaning it could not indicate whether the changes in BRUMS vigor and fatigue were attributed solely to the presence of MF, or other additional factors during training. Similar limitations existed in Selmi et al. (2022), who reported a decrease in BRUMS vigor and increase in fatigue during a 2‐week soccer training camp. Therefore, it appears that there is a tendency for mentally fatigued individuals to rate these two variables negatively as demonstrated in the majority of the MF research (Van Cutsem et al., 2017). The BRUMS confusion rating also presented a small to moderate increase following orienteering training, supporting the consensus in Lam, Sproule, Turner, Murgatroyd, et al., 2023) that confusion can occur in an MF state. Therefore, inclusion of the BRUMS confusion subscale in future MF investigations while measuring the mood state of the orienteers, as well as considering if interventions to reduce MF also effectively reduce ratings of confusion, may be warranted.

#### 4.2.1 Changes in subjective outcome variables 24‐ and 48‐h after orienteering training camp

The ratings for perceived MF, PF, stress, tiredness, BRUMS vigor and confusion remained impaired at 48POST when compared to PRE1, in line with data in soccer players (Thompson et al., 2020; Abbott et al., 2020; Abbott et al., 2018). Inadequate recovery can be related to poor sleep quality during training and competition (Fullagar et al., 2016). However, we did not assess perceived sleep quality and recovery. It remains unclear whether the impaired ratings during and post‐training camp are related to sleep and recovery. Importantly, the aim of this investigation was to observe MF changes during orienteering training. Therefore, future research is needed to examine whether perceived recovery and sleep quality influence the changes in MF.

It is acknowledged that the training camp recorded in this study was a competition preparation camp where the participants were expected to perform a series of simulated races over 4 days instead of conventional orienteering training. Therefore, this study cannot validate whether orienteers would experience the similar delay in recovery observed in this study during a typical preseason training week or camp. Importantly, although previous research has reported that routine school/university education and non‐orienteering challenges might also affect perceived MF and psychological responses negatively (Lam, Sproule, Turner, Murgatroyd, et al., 2023; Thompson et al., 2020), the influence of education on delayed recovery in 24POST and 48POST should be limited as the participants in this study were full‐time students and were on spring break. To address this limitation, future research could implement a longitudinal research design to determine the day‐to‐day changes and observe if a normal training week would generate a similar result to that of a training camp.

## 5 LIMITATIONS

The physical demands of the orienteering competition preparation camp were not recorded; thus, the athlete reports cannot be contextualized to the training load on any particular day. While this study recorded the type of race performed on each specific day of the training camp, it would have been useful to request participants to record their running time and completion time for each orienteering race. This additional information could have facilitated a discussion on whether the duration of the race influenced the extent of changes in self‐report measures, although given that coaches will have been feeding back on performance during the training, this unknown factor may have added further complexity to such interpretation. This study acknowledges that factors such as nutrition intake and sleep quality were not recorded and certainly not controlled, and these can be seen as confounding variables since the recovery status of individuals has been shown to be related to MF (Loch et al., 2020). Additionally, intense training over consecutive days could potentially be detrimental to the recovery process. Furthermore, the study did not record post‐training activities, which could also be considered confounding variables, such as the use of smartphones (Fortes et al., 2019) and playing video games (Faro et al., 2022), which have been shown to impair MF ratings. Participants in this study were all national junior level orienteers, which may not be generalizable to changes in professional orienteers, and further research is required at this elite level. The small sample size in this study is a further limitation, with only 22% of the approximately 50 orienteers who attended the training camp providing both consent and parental consent to participate. It is important to consider that the participants were under the age of 18 years, and parental consent was required before the experiment, which could have further decreased the number of participants due to the delay in submitting relevant documents.

## 6 PRACTICAL APPLICATIONS

This study demonstrates that orienteers can experience MF during orienteering training sessions and emphasizes the importance of addressing this phenomenon in training programmes. Failing to recover from increased perception of MF after previous training days can result in higher perceived MF on subsequent days, which can potentially impact the endurance and cognitive performance of orienteers. To optimize performance and mitigate the accumulation and effects of MF, practitioners are advised to minimize training frequency before competitions. If multiple training sessions are unavoidable, implementing effective fatigue management strategies to enhance recovery is recommended.

## 7 CONCLUSION

This study demonstrated that participating in an orienteering training camp leads to acute increases in ratings of MF, PF, tiredness, BRUMS fatigue and confusion, and a decrease in motivation, stress and BRUMS vigor in national junior orienteers. This study also discovered that perceived MF accumulated during a 4‐day training camp, with post‐training ratings gradually increased until the end of the training camp.

## AUTHOR CONTRIBUTION

Hui Kwan Nicholas Lam: conceptualization, manuscript writing (first draft), participant recruitment, data collection, data analysis, manuscript review and editing. John Sproule: research project supervision, manuscript review and editing; Anthony P. Turner: research project supervision, manuscript review and editing; Shaun M. Phillips: research project supervision, manuscript review and editing.

## CONFLICT OF INTEREST STATEMENT

The authors declare no potential conflict of interest.

## Data Availability

The data are not publicly available due to privacy or ethical restrictions.
